# Continuous Metabolic Monitoring Based on Multi-Analyte Biomarkers to Predict Exhaustion

**DOI:** 10.1038/srep10603

**Published:** 2015-06-01

**Authors:** Michail Kastellorizios, Diane J. Burgess

**Affiliations:** 1 Department of Pharmaceutical Sciences, School of Pharmacy, University of Connecticut, Storrs, CT 06269 U.S.A

## Abstract

This work introduces the concept of multi-analyte biomarkers for continuous metabolic monitoring. The importance of using more than one marker lies in the ability to obtain a holistic understanding of the metabolism. This is showcased for the detection and prediction of exhaustion during intense physical exercise. The findings presented here indicate that when *glucose* and *lactate* changes over time are combined into multi-analyte biomarkers, their monitoring trends are more sensitive in the subcutaneous tissue, an implantation-friendly peripheral tissue, compared to the blood. This unexpected observation was confirmed in normal as well as type 1 diabetic rats. This study was designed to be of direct value to continuous monitoring biosensor research, where single analytes are typically monitored. These findings can be implemented in new multi-analyte continuous monitoring technologies for more accurate insulin dosing, as well as for exhaustion prediction studies based on objective data rather than the subject’s perception.

Metabolic monitoring is the periodic recording of metabolic markers that give information on specific metabolic pathways. Diabetes mellitus, obesity and intense physical activity are a few examples where close metabolic monitoring is necessary^1^,^2^,^3^. This is routinely done in a non-continuous manner, where the individual records their blood glucose, body weight, blood pressure, *etc.* over various periods of time. Technological advances on wearable and/or implantable devices have initiated the transition to real-time, continuous monitoring^4^,^5^,^6^,^7^,^8^. However, monitoring devices have several shortcomings: they trigger the foreign body reaction which results in loss of functionality within a few days, they cannot be implanted directly in the bloodstream, and they typically record only single analytes. The foreign body reaction to implantable medical devices has been shown to be prevented by incorporating drug-eluting biocompatible coatings^8^,^9^,^10^. When a device that is implanted in a peripheral tissue such as subcutaneous tissue is used to monitor analyte changes in the blood, loss of sensitivity and lag times are observed^11^. This is due to the fact that analytes have to diffuse from the bloodstream to the interstitial fluid before being detected.

Exhaustion, also known as fatigue, is the inability of muscle to continue an ongoing physical activity^12^. The perception of exhaustion is highly subjective, and consequently the assessment of a subject’s endurance is currently based on semi-empirical observations that link the fitness and current state (*e.g.* heart rate) of each subject with pre-recorded performance levels^13^,^14^. However, exhaustion has been linked to several metabolic pathways and therefore a close examination of the metabolic processes involved in physical activity could facilitate a more accurate prediction of exhaustion. Depletion of fuel stored in the muscle (*i.e.* creatine and glycogen, that are readily accessible as an energy source), as well as accumulation of metabolic byproducts (*e.g.* chloride and potassium ions and lactic acid) are considered the causes of exhaustion^15^,^16^. These metabolic events take place in the exercising muscle, a body compartment that is not available for real-time, continuous monitoring *via* implantable devices mainly due to the trauma associated with intramuscular implantations.

In this work, exhaustion detection in the subcutaneous tissue using multiple analytes combined into multi-analyte biomarkers was investigated. Single-analyte monitoring, such as glucose monitoring, creates blind spots in the recorded metabolic state since a single analyte cannot account for all pathways involved in a given metabolic event. It is hypothesized that monitoring of two analytes (glucose and lactate) will offer a more complete view into metabolic changes during exercise that lead up to exhaustion. In the context of exhaustion prediction, glucose and lactate are suitable candidates for multi-analyte monitoring: glucose is the primary energy source of muscle cells, and lactate is the main by-product of anaerobic metabolism during intense activity.

To test the above hypothesis, exercise experiments were conducted in normal as well as type 1 diabetic rats. Diabetic rats were included in the study design since one application of this work will be to improve the health of diabetic athletes. External observations (commencement of exercise, running speed, and onset of exhaustion) were correlated with internal shifts in metabolism (glucose and lactate as single readings or combined into multi-analyte biomarkers) recorded in the subcutaneous tissue and the blood. Microdialysis was used to monitor glucose and lactate in the subcutaneous tissue and the blood. Microdialysis is a reliable technique that has been used extensively in laboratory settings to monitor analyte changes in various tissues.

## Results

### Choice of analytes to be monitored

**Figure 1:**
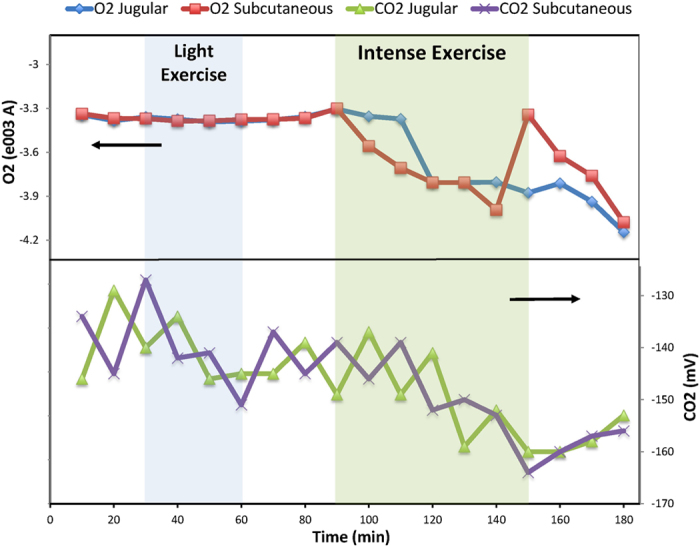
Effect of light and intense exercise on oxygen and carbon dioxide levels in the blood and subcutaneous tissue. Areas shaded blue and green indicate light and intense exercise, respectively, and unshaded areas indicate periods of inactivity (rest or recovery). The top panel displays oxygen level changes and the bottom panel carbon dioxide changes in the dialysate. Arrows indicate y axis. Oxygen levels did not respond to light exercise but only during intense exercise. Carbon dioxide levels responded to both light and intense exercise, but showed highly unstable readings.

Glucose and lactate were chosen as markers based on preliminary studies where blood and subcutaneous glucose, lactate, oxygen and carbon dioxide were monitored in healthy and obese animals during light exercise. The results indicated that oxygen and carbon dioxide are not suitable analytes for predicting exhaustion. As shown in Fig. 1, oxygen trends did not respond to light exercise while carbon dioxide values were highly unstable.

### Glucose and lactate changes during intense exercise

**Figure 2:**
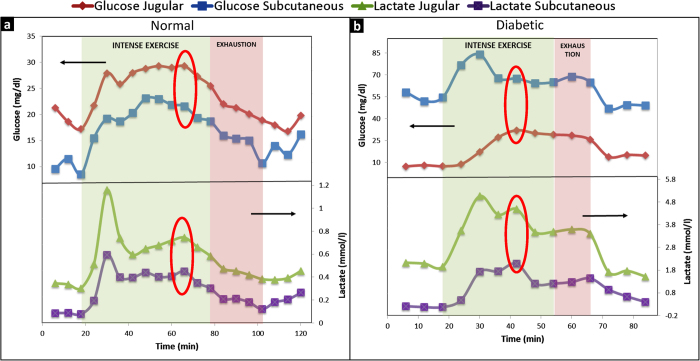
Effect of intense exercise and exhaustion on glucose and lactate levels in the blood and subcutaneous tissue. Areas shaded green and red indicate periods of intense exercise and exhaustion, respectively. Exhaustion was defined as the time when the animal could not keep up with the exercise and the running speed needed to be adjusted. Unshaded areas indicate periods of inactivity (rest or recovery). Arrows indicate y axis. Top panels show glucose and lower panels show lactate changes in the dialysate. Panel *a* shows representative graph from a normal rat and panel *b* from a diabetic rat. Changes in analyte trends precede the onset of exhaustion (red ovals). These are clearer in lactate trends for both normal and diabetic rats.

Figure 2a depicts a representative metabolic profile for a healthy rat. Glucose and lactate changes in the blood and subcutaneous tissue are shown in four zones. *A:* prior to exercise (baseline measurements); *B:* during intense exercise; *C:* during light exercise (post-exhaustion); and *D:* recovery. Since glucose and lactate have to diffuse from the blood to the subcutaneous tissue to be taken up by the microdialysis catheter, the small differences in blood and subcutaneous values were expected. A spike in both glucose and lactate levels in the blood as well as the subcutaneous tissue are observed upon commencement of exercise, a typical observation when transitioning from rest to activity. Exhaustion was first observed 57 minutes into the exercise, when the animal could not keep up and the treadmill speed had to be adjusted. Full exhaustion was observed 30 minutes later, when the animal was unable to continue the exercise. Interestingly, glucose and lactate showed a trend change around 45 minutes into the exercise, 12 minutes before the first physical evidence of exhaustion and 42 minutes before full exhaustion (indicated by red ovals). The exhaustion-prediction window varied from 12 to 20 minutes among the rats, due to varied endurance levels. The average exhaustion prediction time was 16.17 ± 4.16 min.

Similar observations of exhaustion prediction were made in untreated, type 1 diabetic rats (Fig. 2b) with an average exhaustion prediction time of 8.60 ± 3.08 min. However, diabetic rats did not show a gradual manifestation of exhaustion, but reached full exhaustion immediately. As shown in Fig. 2b, glucose as well as lactate build-up was observed. The type 1 diabetic rats were untreated (they did not receive insulin injections). As a result, glucose released from the liver as an energy source could not be internalized by muscle cells which failed to perform the exercise shortly after glucose and lactate build-up stopped. It should be noted that the data from diabetic rats exhibited one anomaly: the glucose values obtained from the microdialysis samples were consistently lower than the values obtained from normal rats. This conflicted with glucose values obtained *via* tail vein pricking to confirm the diabetic state (>350 mg/dL). However, the interpretation of the data shown here relies on the trend rather than the absolute values of glucose and lactate, and this anomaly did not interfere with data interpretation.

The findings described above confirmed our hypothesis that detectable metabolic changes precede physical manifestations of exhaustion. Accordingly, these changes may be used as a predictive tool for exercise endurance and exhaustion.

### Multi-analyte biomarker identification

**Figure 3:**
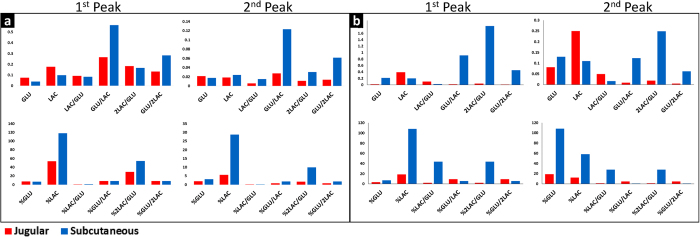
Slope changes for various biomarker combinations of glucose (GLU) and lactate (LAC). All ratios are molar and the percentile changes are calculated based on the baseline measurements before the commencement of the exercise. The first peak represents metabolic changes from rest to activity and the second peak metabolic changes that precede exhaustion (predictive). Panel *a* shows representative results from a normal rat and panel *b* from a diabetic rat.

The observations described above are based on blood levels of glucose and lactate (monitored independently) as these changes were not as evident in the subcutaneous tissue. This poses a potential problem in translating these findings into subcutaneously-implantable monitoring platforms. It was hypothesized that by combining glucose and lactate readings into multi-analyte biomarkers it may be possible to increase the monitoring resolution in the subcutaneous tissue since glucose and lactate account for aerobic and anaerobic metabolism, respectively. Glucose (GLU) and lactate (LAC) combinations were investigated. Since one glucose molecule yields two lactate molecules, the lactate concentration was used directly or multiplied by two to account for this. The efficacy of the biomarkers to increase the monitoring resolution in the subcutaneous tissue was tested by measuring the absolute slope changes during two metabolic events: rest-to-activity transition (first peak in Fig. 2a and 2b) and pre-exhaustion trend changes (second peak in Fig. 2ab). It was determined that combinations of biomarkers were several times more sensitive to metabolic changes in the subcutaneous tissue compared to the blood as shown in Fig. 3a for normal rats and Fig. 3b for diabetic rats. Single analyte changes relative to the baseline measurements (%GLU and %LAC) also showed higher sensitivity in the subcutaneous tissue than the blood. The utilization of percent changes of single analytes is not feasible for continuous monitoring systems as stable baseline measurements are not usually available outside controlled laboratory environments. This is especially relevant for individuals undergoing fitness level changes (due to training or disease management) whose metabolic baseline is continuously shifting, as well as subjects with no control over their activity levels such as deployed military personnel.

### Multi-analyte biomarkers for prediction of exhaustion

**Figure 4:**
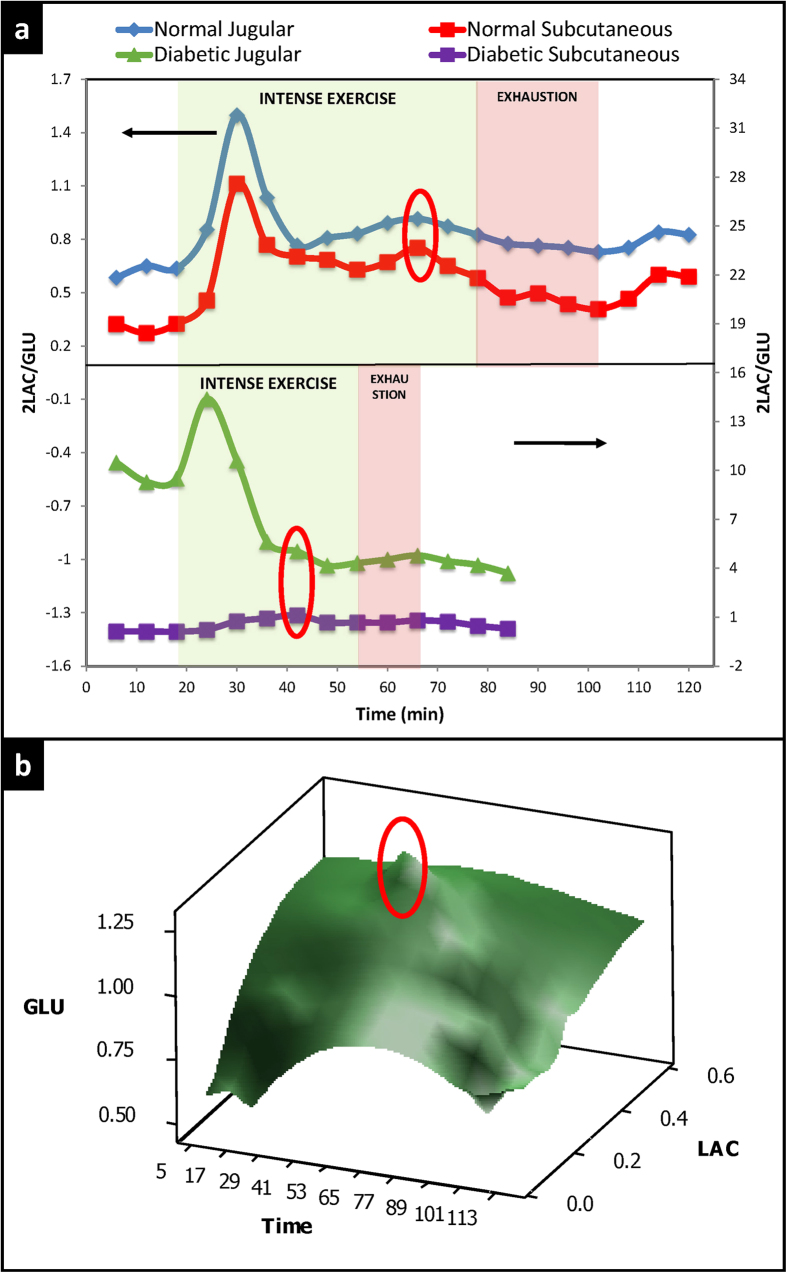
(**a**) Effect of intense exercise and exhaustion on the biomarker *2LAC/GLU* (molar ratio of lactate and glucose multiplied by 2). Areas shaded green and red indicate periods of intense exercise and exhaustion, respectively. Exhaustion was defined as the time when the animal could not keep up with the exercise and the running speed needed to be adjusted. Unshaded areas indicate periods of inactivity (rest or recovery). Arrows indicate y axis. Red ovals indicate the exhaustion-prediction point. The top panel shows representative results from a normal rat and the bottom panel from a diabetic rat. 2LAC/GLU is the optimum biomarker for detecting metabolic changes in the subcutaneous tissue predictive of imminent exhaustion. The results are less clear in the diabetic rat; please note that diabetic rats did not receive insulin treatment, and the baseline glucose was high. This likely interfered with the biomarkers. (**b**) 3D representation of glucose and lactate changes during intense exercise and exhaustion in a normal rat. This plot reveals the interdependence of glucose and lactate that can be extrapolated to evaluate the metabolic flexibility (the ability to transition from aerobic to anaerobic energy utilization). The red oval indicates metabolic changes predictive of exhaustion.

Based on the results shown in Fig. 3a and 3b, *2LAC/GLU* was plotted *vs.* time for normal and diabetic rats (Fig. 4a). As shown in this figure, *2LAC/GLU* can be used to predict exhaustion with more accuracy in the subcutaneous tissue than the blood. 3D surface plots of glucose, lactate, and time were constructed (Fig. 4b). These plots incorporate all possible combinations of glucose and lactate and when implemented into standardized exercise routines^17^,^18^,^19^,^20^,^21^,^22^,^23^,^24^,^25^,^26^,^27^ can provide feedback on the fitness and endurance of the subject. As shown in Fig. 4b, the exhaustion-predictive metabolic changes described above in response to exercise are more profound in areas where glucose and lactate are combined compared to the peripheral regions of the graph where glucose and lactate are plotted separately.

## Discussion

This work studied multi-analyte monitoring in the subcutaneous tissue, an implantation-friendly space for monitoring devices. So far, continuous metabolic monitoring based on implantable biosensors relies on single analytes, usually glucose. The choice of glucose as a monitoring marker is justified by its central role in energy metabolism, as well as its importance in metabolic disorders such as diabetes and obesity. Glucose monitoring alone, however, is bound to suffer from blind spots, despite glucose’s central role in energy metabolism. Moreover, the time it takes for glucose to passively diffuse between the subcutaneous tissue and the blood is known as the lag time and it creates delays in the monitoring curves. These delays, typically in the range of 5 to 15 minutes, are of importance to acute episodes such as hypoglycemia and energy exhaustion in the exercising muscle.

It was hypothesized here that monitoring of multiple analytes at once will increase the monitoring sensitivity and overcome some of the obstacles posed by single-analyte monitoring. Continuous monitoring of more than one analyte can reveal the interdependence of different metabolic pathways and the metabolic flexibility of the individual (the ability to switch from aerobic to anaerobic utilization of energy and vice versa). It was determined that glucose and lactate, when monitored simultaneously, produce metabolic monitoring curves with higher sensitivity in the subcutaneous tissue compared to the blood. This is a significant breakthrough for subcutaneously-implanted monitoring devices since until now their inability to detect analytes directly in the blood was considered a handicap. In addition, the data presented here provide proof-of-concept for multi-analyte biomarkers to be utilized in predictive algorithms. Experiments in exercising animals showed that metabolic changes recorded by multi-analyte monitoring preceded the onset of exhaustion, defined as the point when the animal could not keep up the exercise at the maximum intensity. The development of predictive algorithms was not attempted at this time, however, since data from implantable, multi-analyte biosensors are required for the appropriate statistical analysis. However, all animals studied here exhibited this phenomenon of metabolic changes preceding exhaustion, albeit with different time windows. This variability is likely due to inter- and intra-animal variations in performance levels and indicates that future devices depending on multi-analyte monitoring to predict exhaustion will rely on individual calibration routines.

The ultimate goal is to utilize the proof-of-concept presented here to develop exhaustion-predicting algorithms that rely on holistic metabolic shifts instead of single markers such as heart rate or lactate. The size and shape of the semi-implantable microdialysis probe used here makes it a good model for implantable biosensors. In the future, when implantable multi-sensors become available, the concept of continuous multi-analyte monitoring for exhaustion prediction presented here will be applied to individualized algorithms paired with such devices.

The potential applications of this concept are many. Long-term changes in multi-analyte biomarker interdependence are expected to reveal progress in the training of athletes, management of diabetic patients, development of pre-diabetes in obese subjects, *etc.* Here it was shown that multi-analyte biomarkers may be used to predict exhaustion, which is crucial for diabetic athletes, deployed military personnel, and other high-risk individuals involved in intense physical activity.

## Conclusions

Multi-analyte monitoring was successfully studied to monitor metabolic changes in the subcutaneous tissue during physical exercise in rats. The resolution of such monitoring in the subcutaneous tissue was higher compared to the blood, which is of great importance since monitoring devices cannot be implanted directly in the bloodstream. Simultaneous glucose and lactate monitoring was superior to either glucose or lactate single-analyte monitoring, in terms of the ability to detect metabolic changes in the subcutaneous tissue. In addition, the potential of multi-analyte monitoring to be used to predict exhaustion was confirmed by providing proof-of-concept. The present work demonstrates the need for implantable multi-analyte biosensors. Such biosensors can be used to expand the proof-of-concept provided here to develop exhaustion prediction algorithms.

## Methods

### Animal models

Male Sprague Dawley rats (6 weeks old, 150–170 g) were used as the normal animal model (n = 6). One group of rats was injected with streptozotocin (60 mg/kg body weight, IP) to induce type 1 diabetes (n = 3). All animal studies were done in accordance with the approved guidelines. All studies were reviewed and approved by the University of Connecticut’s Institutional Animal Care and Use Committee (IACUC) prior to the beginning of the experiments.

### Microdialysis

Microdialysis was used to monitor analytes in the blood and subcutaneous tissue. Each rat was implanted with one microdialysis catheter (20 kDa molecular weight cut-off, CMA Microdialysis AB) in the subcutaneous tissue and one in the jugular vein. Catheterization took place with the aid of a guiding needle as per the manufacturer’s instructions.

### Exercise experiments

Rats implanted with two microdialysis catheters were placed in a forced exercise treadmill (IITC, Inc.). An isotonic liquid (Ringer’s solution) was pumped through the microdialysis catheters at 5 μl/min speed rate (syringe pump, Harvard Apparatus) and samples were collected every 10 minutes (preliminary studies) and every 6 minutes for the all other studies. After an initial resting period to collect baseline data, exercise commenced at maximum running speed. The maximum speed varied with each rat and was in the range of 15–17 meters per minute. The onset of exhaustion was noted when the rats could not keep up at the maximum pace, and the exercise ended when the rats failed to run at all. A recovery period was allowed after the exercise.

### Analyte quantification

YSI 2300 STAT Plus™ (YSI Life Sciences, Inc.) was used to quantify glucose and lactate molar concentrations in the microdialysis samples. Oxygen and carbon dioxide microelectrodes (Microelectrodes, Inc.) were used to determine analyte trends in the microdialysis samples. Since oxygen and carbon dioxide monitoring was found unsuitable for this application, only the electrode output is reported to obtain the analyte trend.

### Data analysis

Analyte concentrations were plotted against time as shown in the main text of the manuscript. With the exception of the exhaustion prediction times (which are reported as average values ± standard deviation), animal data could not be aggregated for statistical analysis due to variability on the fitness and endurance level of each rat. Representative plots of analyte trends are shown to showcase the feasibility of multi-analyte biomarkers for exhaustion prediction.

## Additional Information

**How to cite this article**: Kastellorizios, M. and Burgess, D. J. Continuous Metabolic Monitoring Based on Multi-Analyte Biomarkers to Predict Exhaustion. *Sci. Rep.* **5**, 10603; doi: 10.1038/srep10603 (2015).
